# Comparative Study of Flavonoid Profiles, Antioxidant, and Antiproliferative Activities in Hot-Air and Vacuum Drying of Different Parts of Pitaya (*Hylocereus undatus* Britt) Flowers

**DOI:** 10.3390/antiox13080956

**Published:** 2024-08-07

**Authors:** Caifeng Shi, Huaqian Long, Jia Hu, Xinbo Guo

**Affiliations:** School of Food Science and Engineering, South China University of Technology, Guangdong Province Key Laboratory for Green Processing of Natural Products and Product Safety, Engineering Research Center of Starch and Vegetable Protein Processing Ministry of Education, Guangzhou 510640, China; feshicaifeng@mail.scut.edu.cn (C.S.); 202166310591@mail.scut.edu (H.L.); 18582285921@163.com (J.H.)

**Keywords:** pitaya flowers, flavonoid profiles, antioxidant activities, antiproliferation

## Abstract

Pitaya flower, a medicinal and edible plant commonly used in tropical and subtropical regions, was the focus of this study, which compared the effects of hot-air drying (HAD) and vacuum drying (VD) on phytochemical profiles and biological activities of its four parts: calyx, petals, stamens, and pistils. Both drying methods significantly increased the total phenolic content (TPC) of pitaya flowers, with values ranging from 1.86 to 3.24 times higher than those of fresh samples. Twelve flavonoid compounds were identified in pitaya flowers, with the glycoside derivatives of three flavonols (kaempferol, isorhamnetin, and quercetin) being the most abundant. VD resulted in 1.15 times higher total flavonoid glycoside content than HAD, whereas in petals, HAD yielded a total flavonoid glycoside content 1.21 times higher than VD. Both HAD and VD effectively increased the antioxidant capacities of pitaya flowers, though the difference between the two methods was not significant. Additionally, both drying methods enhanced the antiproliferative activity of pitaya flowers, with HAD showing a more significant effect than VD. The present study emphasized the efficacy of drying methods for enhancing flavonoids in pitaya flowers and provided insights for functional products’ innovation with different parts of pitaya flowers.

## 1. Introduction

Pitaya (*Hylocereus undatus* Britt), belonging to the Cactaceae family, is a medicinal and edible plant commonly found in tropical and subtropical regions. Documented for the first time in the “Lingnan Herbal Collection”, pitaya flowers have been traditionally used in Chinese medicine for their properties in clearing heat and phlegm, relieving pain, and regulating phlegm [1]. In southern China, these flowers are popular ingredients in soups and vegetable dishes due to their tonic and maintenance properties. In addition to essential amino acids, vitamins, unsaturated fatty acids, and other nutrients, pitaya flowers also contain active phytochemicals, such as polyphenols, flavonoids, and polysaccharides, which have antioxidant, anti-inflammatory, hypolipidemic, and anti-tumor beneficial effects [2]. The polysaccharides derived from pitaya flowers, for instance, have shown promising antioxidant and immunomodulatory properties, suggesting their potential for therapeutic applications [3]. Furthermore, aqueous extracts of pitaya flowers have been found to alleviate colitis by restoring the integrity of the intestinal barrier, reducing inflammation, and modulating the intestinal and lung microbiota [4]. This therapeutic potential underscores the importance of exploring the phytochemical composition of pitaya flowers to fully understand their benefits. In recent years, polyphenols have garnered significant attention as key active components of pitaya flowers. Previous studies have identified major polyphenolic constituents in pitaya flowers: kaempferol, quercetin, and isorhamnetin, along with their glycoside analogs [5]. These compounds are known for their antioxidant properties, which can neutralize harmful free radicals and protect against oxidative-stress-related diseases [6]. However, the major components and polyphenol content varied among the four parts of the calyx, petals, stamens, and pistils [7]. This variation highlights the need for targeted research to assess which parts of the flower are richest in beneficial compounds and to understand how these components contribute to the overall health benefits of the flower.

In pitaya cultivation, flower thinning is commonly performed to enhance fruit quality, resulting in a substantial amount of agricultural waste [8]. Rational development and utilization of pitaya flowers can reduce disposal costs and increase their added value. Fresh pitaya flowers are highly perishable due to their high moisture content and elevated polyphenol oxidase and peroxidase activities. Therefore, drying is an effective method for extending their storage period. However, research on the drying methods for the calyx, petals, stamens, and pistils of pitaya flowers is scarce. The choice of drying method can significantly influence the retention of bioactive compounds and the overall quality of the dried flowers. Consequently, exploring drying methods to maintain the nutritional quality and medicinal value of pitaya flowers is crucial for their industrial application.

Currently, hot-air drying (HAD) and vacuum drying (VD) are widely used in the industrial production of agricultural products due to their simple equipment requirements and versatile energy use. HAD is fast and efficient, but the complete exposure of samples to air and high temperatures can cause non-enzymatic browning, such as the Maillard reaction, which reduces color quality [9]. Conversely, VD effectively isolates oxygen and protects the samples, but it is time-consuming and costly [10]. Therefore, this study aimed to determine the effects of HAD and VD on the phytochemical composition, antioxidant activity, and antiproliferative activity of different parts of pitaya flowers. The examination of individual parts of flowers allows for a more nuanced understanding of the distribution and concentration of bioactive compounds. By identifying specific flower parts with higher levels of beneficial phytochemicals, we can better target these components for therapeutic use and further studies. These findings will guide efforts to enhance the bioactivity and drying efficiency of pitaya flowers during the dehydration process, which is crucial for their industrial application and may help increase their value while reducing agricultural waste.

## 2. Materials and Methods

### 2.1. Sample Preparation

The variety of pitaya flowers used in this study was ‘Jindu No. 1’, collected from a local plantation (Guangzhou, China). Intact and undamaged fresh pitaya flowers were divided into four parts: calyx, petals, stamens, and pistils, and then washed well with tap water. The selected samples were treated with hot-air drying (HAD) at 80 °C and vacuum drying (VD) at 80 °C until they reached a constant weight, then stored with desiccants at room temperature. Fresh pitaya flowers (Fresh) without any drying treatment were used as controls in this study.

### 2.2. Extraction and Determination of Phytochemicals

Based on published protocols, phenolics were extracted following modified laboratory methods [11]. Briefly, 1.00 g of dried sample powder was mixed with 30 mL of 80% acetone (*v*/*v*) and homogenized at 12,000 rpm for 1 min. This homogenization was repeated three times. The homogenate was then centrifuged at 8000 rpm for 10 min, and the supernatant was collected. This extraction process was repeated three times. The combined supernatants were rotary evaporated at 45 °C, dissolved in 80% methanol, and brought to a final volume of 10 mL. The extract was stored at −20 °C until further analysis.

The total phenolic content (TPC) was quantified using the Folin–Ciocalteu method, as previously described [12]. Gallic acid was used as a standard, and the results were expressed as milligrams of gallic acid equivalents per gram of dry weight of the sample (mg GAE/g DW). The polyphenol fractions of pitaya flowers were analyzed qualitatively and quantitatively by high-performance liquid chromatography (HPLC; Waters Corporation, Milford, MA, USA). The analysis was conducted on a C18 column (250 × 4.6 mm, 5 μm) at 360 nm with a column temperature of 25 °C and an injection volume of 20 µL. The gradient elution procedure was as follows: 0–5 min (5% B), 5–40 min (5–25% B), 40–47 min (25–38% B), 47–49 min (38–45% B), 49–51 min (45% B), and 51–55 min (45–5% B). The mobile phase consisted of 0.1% aqueous trifluoroacetic acid (A) and acetonitrile (B) at a flow rate of 0.8 mL/min. Peak times and peak areas were compared with standards for qualitative and quantitative analysis of the polyphenol fractions. Results were expressed as micrograms of polyphenols per gram of dry weight of the sample (μg/g DW, *n* = 3).

### 2.3. Determination of Antioxidant Activities

The total antioxidant capacity of pitaya flowers was evaluated by determining the oxygen radical absorbance capacity (ORAC) and 1,1-diphenyl-2-picrylhydrazyl (DPPH) radical scavenging capacity of the polyphenol extracts. The ORAC method was performed as previously described [13], utilizing water-soluble vitamin E (Trolox) as a standard and calculating the results based on the area under the fluorescence decay curve (AUC) and the concentration of Trolox. The ORAC values were expressed as micromolar Trolox equivalents per gram of dry weight of the sample (μ mol TE/g DW, *n* = 3). The DPPH method was performed using the A153-1-1 kit from the Nanjing Jiancheng Bioengineering Research Institute (Nanjing, China). Absorbance values were measured at 517 nm, and the results were expressed as milligrams of Trolox equivalents per gram of dry weight of the sample (mg Trolox/g DW, *n* = 3).

### 2.4. Determination of Antiproliferative Activity and Cytotoxicity

The antiproliferative and cytotoxic effects of pitaya flower extracts were determined using the methylene blue method [14]. HepG2 (ATCC: HB-8065) cells in DMEM were seeded into 96-well plates at a density of 2.0 × 10^4^ cells/well for antiproliferative activity assays and 2.5 × 10^4^ cells/well for cytotoxicity assays. Following incubation at 37 °C for 4 h or 24 h, respectively, the medium was replaced with solutions containing various concentrations of pitaya flower extract. After further incubation at 37 °C for 72 h (for antiproliferative activity) and 24 h (for cytotoxicity), the medium was discarded, and methylene blue stain was added. Absorbance at 595 nm was measured using an MRX II Dynex plate reader (Dynex Technologies, Inc., Chantilly, VA, USA), and the number of viable cells in each well was calculated using the methylene blue colorimetric method. Antiproliferative activity was expressed as the IC_50_ value (mg/mL DW), and cytotoxicity was expressed as the CC_10_ value (mg/ML DW), with each assay performed in triplicate.

### 2.5. Statistical Analysis

Each experiment was repeated three times, and the results are expressed as the mean ± standard deviation (SD). Concentration effects were calculated using Calcusyn software version 2.0 (Biosoft, Cambridge, UK). Significant differences and correlations between groups were analyzed using one-way analysis of variance (ANOVA) and Duncan’s multiple comparison post hoc test, both conducted with IBM SPSS 25.0 (SPSS Inc., Armonk, NY, USA). A threshold of *p* < 0.05 was set to indicate statistical significance between samples.

## 3. Results

### 3.1. Changes in Total Phenolic Content in Different Parts of Pitaya Flowers after HAD and VD Treatments

**Figure 1:**
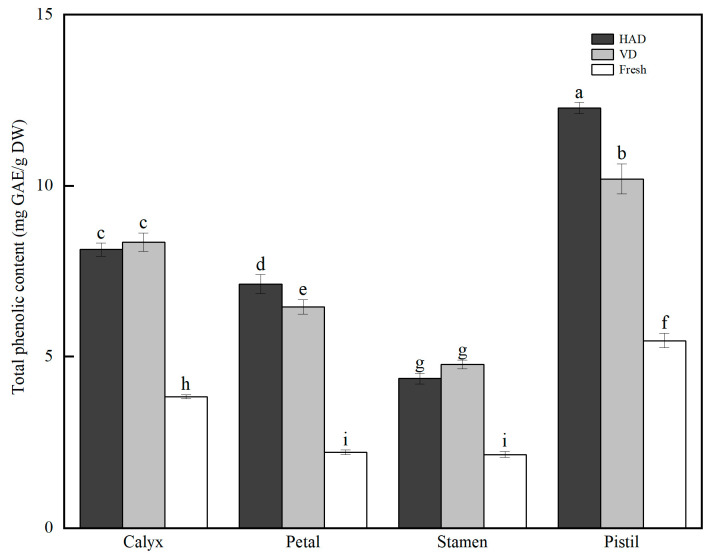
Total phenolic content in different parts of pitaya flowers after HAD and VD treatments (mean ± SD, *n* = 3). Bars with different letters differ significantly at *p* < 0.05.

The total phenolic content (TPC) in different parts of pitaya flowers treated with HAD and VD is presented in Figure 1. In fresh samples, the TPC in pistils was the highest, at 5.47 ± 0.21 mg/g DW, followed by calyx (3.83 ± 0.06 mg/g DW), petals (2.20 ± 0.07 mg/g DW), and stamens (2.14 ± 0.09 mg/g DW). The overall trend of TPC for the four parts in both HAD and VD samples was consistent with that of the fresh samples, ranging from 4.36 ± 0.16 to 12.27 ± 0.16 mg/g DW for HAD and 4.77 ± 0.13 to 10.20 ± 0.44 mg/g DW for VD, respectively. Compared to the fresh samples, both HAD and VD significantly increased the TPC in all four parts of pitaya flowers by 1.86 to 3.24 times. Specifically, compared with VD, HAD improved the TPC in pistils and petals by 20.29% and 10.22%, respectively, while there was no significant difference in the TPC of calyx and stamens between the two drying treatments.

### 3.2. Changes in Flavonoids in Different Parts of Pitaya Flowers after HAD and VD Treatments

Variations in flavonoid constituents of calyx, petals, stamens, and pistils of pitaya flowers under HAD and VD treatments are detailed in Table 1 and Figure S1. A total of twelve phenolic compounds, classified into two categories, flavonoid glycosides and flavones, were found in the free fractions of pitaya flowers using the HPLC technique.

Flavonoid glycosides were the predominant phenolic compounds, categorized into quercetin glycosides (Q3Ru, HY, and Q3G), kaempferol glycosides (K3Rb, K3Ru, and K3G), and isorhamnetin glycosides (I3Rb, I3Ru, and I3G), collectively accounting for 87.50–100.0% of the total phenolic compounds in the samples. In fresh samples, the highest total flavonoid glycosides content was observed in the calyx (4.82 ± 0.21 mg/g DW), followed by petals (3.38 ± 0.22 mg/g DW), stamens (3.09 ± 0.20 mg/g DW), and the lowest in pistils (1.53 ± 0.059 mg/g DW). The total flavonoid glycosides content trends for the four parts in HAD and VD samples closely mirrored those of the fresh samples, with ranges of 2.07 ± 0.07 to 10.30 ± 0.63 mg/g DW for HAD and 1.68 ± 0.04 to 9.97 ± 0.37 mg/g DW for VD, respectively. Compared to fresh samples, both drying methods significantly increased the total flavonoid glycosides content across all parts of pitaya flowers, ranging from 1.10 to 3.05 times higher than fresh samples. Specifically, VD resulted in a total flavonoid glycosides content of 9.97 ± 0.37 mg/g in the calyx, 1.15 times higher than HAD, while HAD yielded higher total flavonoid glycosides content in petals (10.30 ± 0.63 mg/g) compared to VD (8.49 ± 0.52 mg/g), though not statistically significant for stamens and pistils. The nine flavonoid glycoside compounds in the VD calyx ranged from 164.0 ± 5.64 to 3459 ± 119.8 µg/g DW, which were 1.04 to 1.38 times higher than those in the HAD calyx. In contrast, the flavonoid glycoside compounds in VD petals were 17.68% to 45.11% lower than those in HAD petals (144.3 ± 11.25 to 6702 ± 403.7 µg/g DW). Most flavonoid glycoside compounds in the stamens showed no significant difference between the two drying methods. The pistils in VD samples had higher levels of quercetin glycoside compounds (Q3Ru and HY) compared to HAD, but lower levels of kaempferol and isorhamnetin glycoside compounds (K3Ru, I3Rb, I3Ru, and K3G). Among the components of pitaya flowers, kaempferol glycosides and isorhamnetin glycosides were the most prevalent, exhibiting slight variations between parts. All six flavonoid glycosides were identified in the calyx and stamens, while I3G was absent in dried petals. Furthermore, the principal constituents of the pistil did not include K3Rb and I3G.

Three flavonoid aglycones were quantified in the order of QE, KA, and IS. No flavonoids were identified in the fresh pitaya flowers. VD had the highest total flavonoid content in the calyx, followed by the stamen and pistil, and the lowest in the petals, with 435.8 ± 17.72, 264.6 ± 12.48, 236.7 ± 3.78, and 86.66 ± 7.22 μg/g DW, respectively. The total flavonoid content of the four parts of HAD followed the same trend as that of VD and was 1.18–2.08 times higher than that of VD. Compared to the QE, KA, and IS contents of VD calyx (104.4 ± 2.13, 23.24 ± 1.76, and 308.1 ± 14.21 μg/g DW), HAD calyx showed an increase of 54.31%, 90.96%, and 102.95%, respectively. Only KA was detected in petals and stamens, with the HAD sample content (180.6 ± 10.65 and 315.5 ± 31.81 μg/g DW, respectively) being 2.08 and 1.19 times higher than VD. The QE content of the VD pistil (172.2 ± 1.92 μg/g DW) was found to be greater than that of the HAD (123.4 ± 2.61 μg/g DW).

### 3.3. Changes in Antioxidant Activities in Different Parts of Pitaya Flowers after HAD and VD Treatments

The antioxidant activities of pitaya flowers are presented in Table 2, expressed as ORAC and DPPH values. In fresh samples, the highest ORAC values were observed in pistils (15.19 ± 2.50 μmol TE/g DW), followed by calyx (9.20 ± 0.22 μmol TE/g DW), stamens (5.98 ± 0.08 μmol TE/g DW), and petals (5.29 ± 0.17 μmol TE/g DW). The ORAC values of HAD and VD samples for the four parts exhibited similar trends to fresh samples, ranging from 21.63 ± 1.48 to 52.25 ± 7.18 μmol TE/g DW for HAD and 27.93 ± 0.16 to 52.00 ± 6.35 μmol TE/g DW for VD. Both drying methods significantly enhanced the ORAC values of pitaya flowers, increasing them by 2.42 to 6.08 times compared to fresh samples, with no significant difference observed between HAD and VD treatments. 

In fresh samples, the pistils exhibited the highest DPPH values (19.83 ± 1.13 mg Trolox/g DW), followed by the calyx (4.85 ± 0.11 mg Trolox/g DW), with stamens (0.59 ± 0.03 mg Trolox/g DW) and petals (0.60 ± 0.04 mg Trolox/g DW) showing the lowest values. The DPPH values for HAD and VD samples across the four parts followed similar trends to those of the fresh samples, ranging from 5.51 ± 0.30 to 39.16 ± 0.97 mg Trolox/g DW for HAD and 5.47 ± 0.19 to 40.10 ± 1.19 mg Trolox/g DW for VD. Similarly, the non-differential and significant increase in the ability of pitaya flowers to scavenge DPPH free radicals was observed in HAD and VD treatments, with values ranging from 1.97 to 12.23 times higher than those of the fresh samples.

### 3.4. Changes in Antiproliferative Activity and Cytotoxicity in Different Parts of Pitaya Flowers after HAD and VD Treatments

**Figure 2:**
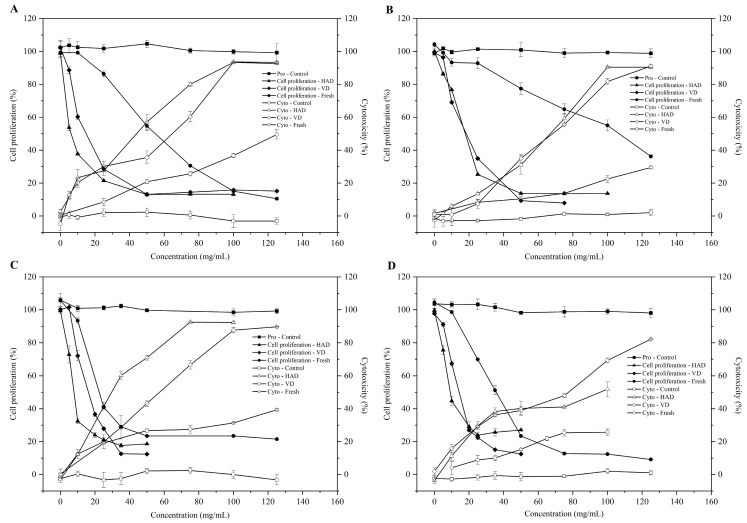
Cytotoxic and antiproliferative activities of extracts from calyx (**A**), petals (**B**), stamens (**C**), and pistil (**D**) of pitaya flowers after HAD and VD treatments toward HepG2 (mean ± SD, *n* = 3).

The effects of HAD and VD treatments on the antiproliferative activity of different parts of pitaya flowers were evaluated using HepG2 cells, as shown in Table 2 and Figure 2. Cytotoxicity was assessed by determining the concentration causing half toxicity to cells (CC_50_), where lower values indicate greater cytotoxicity (Supplementary Table S1). In fresh samples, the calyx and stamens exhibited the highest cytotoxicity, followed by the pistil and petals (CC_50_ values of 98.70 ± 0.63, 95.73 ± 1.74, 112.0 ± 1.90, and 205.5 ± 4.16 mg/mL DW, respectively). The CC_50_ values of HAD and VD samples for the four parts showed similar trends to fresh samples, ranging from 26.78 ± 1.75 to 48.80 ± 3.09 mg/mL DW for HAD and 31.07 ± 1.36 to 62.31 ± 0.43 mg/mL DW for VD. After HAD and VD treatments, the CC_50_ values of pitaya flowers were significantly reduced by 52.31% to 76.25% compared to fresh samples, indicating enhanced cytotoxicity.

Figure 2 illustrates the dose-dependent inhibition of HepG2 cell activity by phytochemical extracts of pitaya flowers. Antiproliferative activity was quantified by the IC_50_ value, where lower values indicate higher antiproliferative activity. In fresh samples, the IC_50_ values for petals, calyx, pistil, and stamen were 65.64 ± 5.12, 53.97 ± 3.39, 37.91 ± 2.00, and 31.03 ± 1.41 mg/mL DW, respectively, with the calyx showing the lowest IC50 value (4.87 ± 0.51 mg/mL DW) and the stamens, pistil, and petals showing 1.61, 2.11 and 3.54 times higher values, respectively. VD samples exhibited IC_50_ values ranging from 14.44 ± 0.16 to 19.12 ± 0.26 mg/mL DW, with significant differences observed only between petals and pistils. The IC_50_ values of fresh pitaya flower samples were 3.69 to 11.08 times higher than those of HAD and 3.08 to 3.96 times higher than those of VD, indicating that both drying methods enhanced the antiproliferative activity of pitaya flowers, with HAD showing a more pronounced effect than VD.

### 3.5. Correlation Analysis

**Figure 3:**
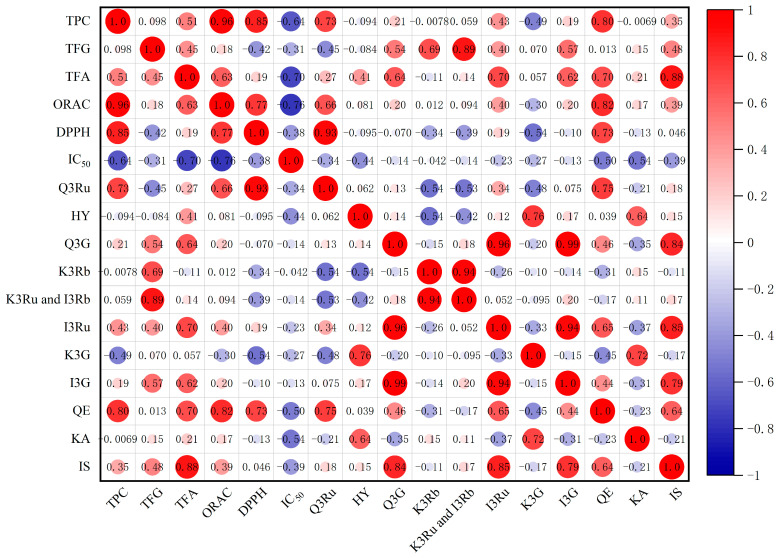
Pearson’s correlation between total phenolic content (TPC), total flavonoid glycosides (TFG), total flavonoid aglycones (TFA), phenolic composition, antioxidant activity (DPPH and ORAC), and antiproliferation (IC_50_) in pitaya flowers.

Pearson’s correlation analysis between phenolics and the antioxidant and antiproliferative activities of pitaya flowers are shown in Figure 3. ORAC values showed significant correlations with total phenolic content (*p* < 0.01), total flavonoid content (*p* < 0.05), and the contents of compounds Q3Ru (*p* < 0.05) and QE (*p* < 0.01). Similarly, DPPH values were closely related to total phenolic content (*p* < 0.01) and the contents of compounds Q3Ru (*p* < 0.01) and QE (*p* < 0.01). Additionally, a strong correlation was observed between ORAC and DPPH values (*p* < 0.01). The antiproliferative activity showed significant associations with total phenolic content (*p* < 0.05) and total flavonoid content (*p* < 0.05). These results indicated that phenolic content in pitaya flowers is positively linked to their antioxidant and antiproliferative activities. Furthermore, significant positive associations were found between the contents of compounds QE and Q3Ru, KA and K3G, IS and I3Ru, and I3G (*p* < 0.01).

## 4. Discussion

### 4.1. Effect of HAD and VD Treatments on the Polyphenol Composition of Pitaya Flowers

In this study, both hot-air drying (HAD) and vacuum drying (VD) significantly increased the total phenolics, total flavonoid glycosides, and total flavone contents in four parts of pitaya flowers—calyx, petals, stamens, and pistils. This enhancement can be attributed to the disruption of cellular structures and possible inactivation of hydrolytic enzymes during dry processing, facilitating the release of phenolics, which are products of plant secondary metabolism [15].

Phytochemical analysis revealed that K3Rb, K3Ru, I3Rb, I3Ru, K3G, and I3G are the predominant constituents of pitaya flowers, albeit with slight variations among different parts. The six compounds were identified in the calyx and stamens, but I3G was not detected in the dried petals. Furthermore, the main constituents of the pistil did not include K3Rb and I3G. This discrepancy with the previous report [7] may be attributed to differences in plant variety, drying methods, and analytical techniques used in the studies. HAD was particularly effective in extracting total phenolic compounds from petals and pistils, while VD excelled with calyxes, though no significant difference was observed for stamens between the two drying methods. These findings suggest that tailored drying treatments are necessary for different parts of pitaya flowers. Similar studies on chrysanthemums have shown that VD enhances TPC in petals and receptacles, with negligible differences observed in stamens between drying methods [16].

Flavone analysis revealed the absence of QE, KA, and IS in fresh pitaya flower samples. Correlation analysis revealed significant positive correlations (*p* < 0.01) between the contents of QE and Q3Ru, KA and K3G, and IS with I3Ru and I3G. These correlations indicate a close association between certain flavonoids and their glycosidic forms. This relationship is consistent with known biochemical pathways, where flavonoid glycosides are typically converted to their aglycone forms through enzymatic processes [17,18]. The higher concentration of flavonoid aglycones in VD calyxes compared to HAD samples could be explained by the differential impact of these drying methods on flavonoid stability. VD maintains the structural integrity of flavonoids and reduces oxidative degradation, leading to higher levels of aglycones. This observation aligns with studies on other plant materials, which showed that vacuum drying better preserves sensitive compounds than traditional hot-air drying [19]. Conversely, the elevated levels of QE, KA, and IS in HAD petals, stamens, and pistils compared to VD suggested that hot-air drying may facilitate the breakdown of glycosidic bonds, thereby releasing these flavonoids. This variation may be attributed to the distribution of PPOs in cellular tissues, influenced by the type, variety, and maturity of the raw material [20], with PPOs predominantly found in plastids, such as chloroplasts and leucoplasts, particularly abundant in young tissues [21]. Consequently, it is hypothesized that pitaya flower calyxes contain higher PPO concentrations, and VD reduces glycosidic bond cleavage in flavones due to oxygen absence, leading to decreased levels of QE, KA, and IS.

### 4.2. Effect of HAD and VD Treatments on Antioxidant Activity of Pitaya Flowers

In this study, ORAC and DPPH assays were employed to assess the antioxidant properties of pitaya flower polyphenols treated with HAD and VD. The ORAC evaluation model utilizes dichlorofluorescin diacetate (DCFH-DA) as a fluorescent probe to assess the interaction between antioxidants and free radicals, which is a rapid and sensitive chemical method for evaluating the scavenging capacity of antioxidants against oxygen free radicals [22]. In the DPPH assay, the lone pair of electrons of the DPPH radical can be paired with antioxidants, and the antioxidant activity of the samples was compared by measuring the absorbance value at 517 nm [23].

As indicated in Table 2, fresh pitaya flower samples exhibited the highest ORAC and DPPH values in pistils, followed by calyx, with stamens and petals showing the lowest values. The results indicate that the pistils had superior antioxidant capacity compared to the other parts, which is consistent with their higher polyphenol content. Both HAD and VD treatments significantly augmented the oxygen and DPPH radical scavenging abilities of pitaya flowers, with no notable difference observed between the two drying methods. This finding suggests that both drying techniques were effective in preserving the antioxidant properties of pitaya flowers, although the choice of method may depend on other practical considerations, such as cost and drying time. Correlation analysis revealed a significant association between ORAC and DPPH values of pitaya flowers and their total polyphenol content (*p* < 0.01), along with the presence of compounds Q3Ru (*p* < 0.05) and QE (*p* < 0.01). This suggests that both flavonoid glycosides and flavone aglycones contributed to radical scavenging by providing phenolic hydroxyl groups. Q3Ru has been highlighted in previous studies as a prominent antioxidant in lychee pulp [24], where it effectively scavenges free radicals and protects cells from oxidative damage [25]. Similarly, QE is known for its potent antioxidant effects through modulation of glutathione levels, enzyme activities, and ROS signaling pathways under environmental stress [26]. Comparable trends have been observed in other fruits and vegetables, where specific flavonoids, particularly glycosides and aglycones, are associated with elevated antioxidant activity [27,28]. These findings underscore the critical role of these flavonoids in bolstering antioxidant defenses, thus supporting the therapeutic potential of pitaya flowers.

### 4.3. Effect of HAD and VD Treatments on Antiproliferative Activity of Pitaya Flowers

Phytochemicals, particularly polyphenols, such as flavones, have been extensively studied for their anti-tumor effects, including modulation of apoptosis, inhibition of migration, and suppression of proliferation [29,30]. Notably, there is a lack of literature on the anti-hepatocellular carcinoma activity of pitaya flower extracts, making this study particularly informative.

As depicted in Figure 2, extracts from different parts of pitaya flowers exhibited varying degrees of HepG2 cell proliferation inhibition. Both HAD and VD treatments enhanced the antiproliferative activity across all four parts of pitaya flowers, with HAD demonstrating superior efficacy over VD. As previously reported in the literature, HAD and VD have been shown to significantly enhance the antiproliferative activity of lily bulbs, which is consistent with the results of this study [31]. Correlation analysis revealed significant associations between antiproliferative activity and total phenolic content (*p* < 0.05), total flavonoid content (*p* < 0.05), and ORAC values. Interestingly, despite lower concentrations, flavonoid alcohols exhibited stronger inhibitory effects against cancer cells, whereas the highest content of flavonoid glycosides showed minimal impact on cell proliferation. This phenomenon may be attributed to the higher bioavailability and cell membrane permeability of flavonoid aglycones, allowing them to more effectively interfere with the physiological functions of cancer cells. In contrast, the glycosylation of flavonoid glycosides reduces their biological activity and effectiveness within cells [32]. The individual contents of compounds QE, KA, and IS did not correlate significantly with IC_50_ values, suggesting that the antiproliferative activity of pitaya flower phytochemicals is likely attributed to synergistic interactions among multiple compounds rather than a single component.

## 5. Conclusions

This study demonstrated that both HAD and VD significantly enhanced total phenolic content (TPC), flavonoid composition, antioxidant capacity, and antiproliferative activity across all parts of pitaya flowers, including the calyx, petals, stamens, and pistil. Among these, the pistil exhibited the highest TPC and demonstrated the strongest scavenging capacity against oxygen and DPPH radicals. Specifically, the HAD-treated calyx showed the highest flavonoid content and exerted the most pronounced antiproliferative effects against HepG2 cells. Moreover, antioxidant activity in pitaya flowers was closely associated with TPC, as well as the contents of compounds Q3Ru and QE, while antiproliferative activity correlated significantly with total flavonoid content. Interestingly, despite lower concentrations, compounds QE, KA, and IS contributed more effectively to inhibiting the proliferation of HepG2 cells. These findings underscore that the antiproliferative effects of pitaya flower extracts resulted from complex interactions among phytochemicals, rather than the singular influence of one or two dominant components. These insights provide a foundation for optimizing drying conditions for pitaya flowers as antioxidant-rich and anticancer dietary supplements in food or cosmetic industries.

## Figures and Tables

**Table 1:** Changes in phenolics in different parts of pitaya flowers after HAD and VD treatments (mean ± SD, *n* = 3).

Compounds and Contents	Parts	Fresh	HAD	VD
Total flavonoid glycosides (mg/g)	Calyx	4.82 ± 0.21 c	8.64 ± 0.20 b	9.97 ± 0.37 a
	Petal	3.38 ± 0.22 d	10.30 ± 0.63 a	8.49 ± 0.52 b
	Stamen	3.09 ± 0.20 d	4.54 ± 0.09 c	4.55 ± 0.08 c
	Pistil	1.53 ± 0.059 f	2.07 ± 0.07 e	1.68 ± 0.04 ef
Q3Ru (μg/g)	Calyx	69.73 ± 4.89 g	235.0 ± 8.88 e	264.9 ± 9.46 d
	Petal	ND	ND	ND
	Stamen	39.39 ± 3.66 h	102.6 ± 1.38 f	104.9 ± 4.89 f
	Pistil	394.0 ± 29.19 c	438.9 ± 26.92 b	596.1 ± 13.75 a
HY (μg/g)	Calyx	98.04 ± 4.16 e	156.6 ± 3.20 c	164.0 ± 5.64 c
	Petal	ND	ND	ND
	Stamen	118.6 ± 5.09 d	311.0 ± 7.09 b	331.4 ± 10.56 a
	Pistil	67.67 ± 6.59 g	80.83 ± 2.47 f	104.7 ± 6.91 h
Q3G (μg/g)	Calyx	169.1 ± 13.22 c	362.9 ± 0.91 b	425.7 ± 12.00 a
	Petal	ND	ND	ND
	Stamen	ND	ND	ND
	Pistil	62.34 ± 0.42 d	ND	ND
K3Rb (μg/g)	Calyx	178.2 ± 8.93 e	387.5 ± 9.60 d	459.5 ± 19.31 d
	Petal	796.0 ± 36.98 c	2641 ± 162.6 a	2174 ± 133.9 b
	Stamen	89.43 ± 5.18 ef	135.8 ± 2.79 e	154.0 ± 4.35 e
	Pistil	0.56 ± 0.03 f	ND	ND
K3Ru and I3Rb (μg/g)	Calyx	1825 ± 57.06 f	3141 ± 66.89 d	3459 ± 119.8 c
	Petal	2116 ± 178.9 e	6702 ± 403.7 a	5609 ± 350.1 b
	Stamen	775.3 ± 47.98 h	1146 ± 22.22 g	1214 ± 35.28 g
	Pistil	375.0 ± 28.03 ij	582.4 ± 19.88 hi	406.6 ± 10.76 j
I3Ru (μg/g)	Calyx	1228 ± 42.44 c	2333 ± 55.77 b	2437 ± 109.1 a
	Petal	70.80 ± 4.48 j	144.3 ± 11.25 gh	79.21 ± 6.48 hi
	Stamen	105.1 ± 2.97 ghi	139.1 ± 12.49 ghi	172.2 ± 4.97 g
	Pistil	547.2 ± 39.44 f	918.2 ± 21.68 d	581.9 ± 13.99 e
K3G (μg/g)	Calyx	418.7 ± 35.24 f	559.3 ± 12.61 e	748.6 ± 27.76 d
	Petal	372.6 ± 0.78 f	813.7 ± 49.98 d	634.5 ± 30.90 e
	Stamen	1936 ± 135.5 c	2669 ± 52.87 a	2532 ± 40.11 b
	Pistil	85.44 ± 2.20 g	49.07 ± 5.25 g	ND
I3G (μg/g)	Calyx	829.7 ± 90.43 c	1461 ± 41.30 b	2019 ± 82.89 a
	Petal	24.28 ± 0.15 d	ND	ND
	Stamen	27.76 ± 1.77 d	44.25 ± 2.12 d	36.81 ± 3.08 d
	Pistil	ND	ND	ND
Total flavonoid aglycones (μg/g)	Calyx	ND	830.8 ± 40.47 a	435.8 ± 17.72 b
	Petal	ND	180.6 ± 10.65 f	86.66 ± 7.22 g
	Stamen	ND	315.5 ± 47.84 c	264.6 ± 12.48 de
	Pistil	ND	280.3 ± 5.40 d	236.7 ± 3.78 e
QE (μg/g)	Calyx	ND	161.1 ± 7.21 b	104.4 ± 2.13 d
	Petal	ND	ND	ND
	Stamen	ND	ND	ND
	Pistil	ND	123.4 ± 2.61 c	172.2 ± 1.92 a
KA (μg/g)	Calyx	ND	44.38 ± 0.39 e	23.24 ± 1.76 f
	Petal	ND	180.6 ± 10.65 c	86.66 ± 7.22 d
	Stamen	ND	315.5 ± 31.81 a	264.6 ± 12.48 b
	Pistil	ND	70.87 ± 1.85 d	64.49 ± 1.87 de
IS (μg/g)	Calyx	ND	625.3 ± 41.14 a	308.1 ± 14.21 b
	Petal	ND	ND	ND
	Stamen	ND	ND	ND
	Pistil	ND	86.08 ± 1.14 c	ND
Total phenolics (mg/g)	Calyx	4.82 ± 0.21 d	9.47 ± 0.19 b	10.41 ± 0.39 a
	Petal	3.38 ± 0.22 e	10.48 ± 0.64 a	8.58 ± 0.52 c
	Stamen	3.09 ± 0.20 e	4.86 ± 0.07 d	4.81 ± 0.10 d
	Pistil	1.53 ± 0.06 g	2.35 ± 0.07 f	1.92 ± 0.02 fg

Means with different letters in a row are significantly different at *p* < 0.05. ND: Not Detected. Q3Ru: quercetin-3-o-rutinoside, HY: hyperoside, Q3G: quercetin-3-o-glucoside, K3Rb: kaempferol-3-o-rubinobioside, K3Ru: kaempferol-3-o-rutinoside, I3Rb: isorhamnetin-3-o-rubinobioside, I3Ru: isorhamnetin-3-o-rutinoside, K3G: kaempferol-3-o-glucoside, I3G: isorhamnetin-3-o-glucoside, QE: quercetin, KA: kaempferol, IS: isorhamnetin.

**Table 2:** Changes in antioxidant and antiproliferative activities in different parts of pitaya flowers after HAD and VD treatments (mean ± SD, *n* = 3).

Parts	Treatments	Antioxidant Activities		Antiproliferation IC_50_ (mg/mL DW)
		DPPH Value (mg Trolox/g DW)	ORAC Value (μmol TE/g DW)	
Calyx	Fresh	4.85 ± 0.11 g	9.20 ± 0.22 f	53.97 ± 3.39 b
	HAD	11.62 ± 0.53 d	40.40 ± 4.07 bc	4.87 ± 0.51 h
	VD	12.10 ± 0.05 d	38.87 ± 4.46 c	17.52 ± 0.91 ef
Petal	Fresh	0.60 ± 0.04 h	5.29 ± 0.17 f	65.64 ± 5.12 a
	HAD	7.34 ± 0.70 e	32.17 ± 1.03 cd	17.25 ± 0.23 ef
	VD	5.47 ± 0.19 fg	28.83 ± 2.28 de	19.12 ± 0.26 e
Stamen	Fresh	0.59 ± 0.03 h	5.98 ± 0.08 f	31.03 ± 1.41 d
	HAD	5.51 ± 0.30 fg	21.63 ± 1.48 e	7.84 ± 0.63 gh
	VD	5.85 ± 0.45 f	27.93 ± 0.16 de	16.16 ± 0.39 ef
Pistil	Fresh	19.83 ± 1.13 c	15.19 ± 2.50 f	37.91 ± 2.00 c
	HAD	39.16 ± 0.97 b	52.25 ± 7.18 a	10.28 ± 1.02 g
	VD	40.10 ± 1.19 a	52.00 ± 6.35 a	14.44 ± 0.16 f

Means with different letters in a row are significantly different at *p* < 0.05.

## Data Availability

Data are available from the authors upon reasonable request.

## Supplementary Materials

The following supporting information can be downloaded at: https://www.mdpi.com/article/10.3390/antiox13080956/s1. Table S1: Changes in cytotoxicity in four parts of pitaya flowers after HAD and VD treatments (mean ± SD, *n* = 3). Figure S1: Chromatograms of extracts from calyx (A), petals (B), stamens (C), and pistils (D) of pitaya flowers after HAD and VD treatments. Notes for Figure S1: 1: quercetin-3-o-rutinoside (Q3Ru), 2: hyperoside (HY), 3: quercetin-3-o-glucoside (Q3G), 4: kaempferol-3-o-rubinobioside (K3Rb), 5 and 6: kaempferol-3-o-rutinoside (K3Ru) and isorhamnetin-3-o-rubinobioside (I3Rb), 7: isorhamnetin-3-o-rutinoside (I3Ru), 8: kaempferol-3-o-glucoside (K3G), 9: isorhamnetin-3-o-glucoside (I3G), 10: quercetin (QE), 11: kaempferol (KA), and 12: isorhamnetin (IS).

## Abbreviations

HAD: hot-air drying; VD: vacuum drying; TPC: total phenolic content; Q3Ru: quercetin-3-o-rutinoside; HY: hyperoside; Q3G: quercetin-3-o-glucoside; K3Rb: kaempferol-3-o-rubinobioside; K3Ru: kaempferol-3-o-rutinoside; I3Rb: isorhamnetin-3-o-rubinobioside; I3Ru: isorhamnetin-3-o-rutinoside; K3G: kaempferol-3-o-glucoside; I3G: isorhamnetin-3-o-glucoside; QE: quercetin; KA: kaempferol; IS: isorhamnetin.
