# Supplementary material for: Comparative Study of Flavonoid Profiles, Antioxidant, and Antiproliferative Activities in Hot-Air and Vacuum Drying of Different Parts of Pitaya (Hylocereus undatus Britt) Flowers
Source: Antioxidants (Basel). 2024 Aug 7;13(8):956. doi: 10.3390/antiox13080956 (PMC11351529; doi:10.3390/antiox13080956)
Supplement: Supplementary file 1 [file antioxidants-13-00956-s001.zip › Table S1.pdf]

Table S1. Changes of cytotoxicity in four parts of pitaya flowers after HAD and VD treatments (mean ± SD, n = 3).

Parts	Treatments	Cytotoxicity (mg/mL DW)		
		CC_{10}	CC_{20}	CC_{50}
Calyx	Fresh	36.28±1.73b	53.52±1.56b	98.70±0.63c
	HAD	6.01±0.50g	10.43±0.80g	26.78±1.75h
	VD	6.16±0.16g	11.20±0.25g	31.07±1.36g
Petal	Fresh	42.47±3.27a	75.97±4.22a	205.5±4.16a
	HAD	15.92±2.22e	24.06±2.65d	48.80±3.09f
	VD	29.72±1.01c	39.06±0.94c	62.31±0.43d
Stamen	Fresh	25.48±0.68d	41.53±0.89c	95.73±1.74c
	HAD	9.66±0.26fg	14.10±0.30fg	26.92±0.36h
	VD	12.88±1.60e	20.54±2.08d	45.65±2.96f
Pistil	Fresh	31.17±6.17c	49.80±5.83b	112.0±1.90b
	HAD	10.45±1.50f	18.35±1.95ef	48.21±2.31f
	VD	9.04±1.19fg	17.39±1.65ef	53.26±1.98e

Means with different letters in a row are significantly different at $p < 0.05$.
